# Phase relations in the Nb–Ni–Cr system at 1,100 °C

**DOI:** 10.1007/s00706-012-0750-4

**Published:** 2012-08-25

**Authors:** Alexander A. Kodentsov, Frans J. J. van Loo

**Affiliations:** Laboratory of Materials and Interface Chemistry, Eindhoven University of Technology, P.O. Box 513, 5600 MB Eindhoven, The Netherlands

**Keywords:** Solid state, Alloys, Phase diagrams, Transition metals compounds

## Abstract

**Abstract:**

The isothermal cross section through the ternary phase diagram Nb–Ni–Cr at 1,100 °C was constructed by means of diffusion couples and equilibrated alloys. It was found that nearly 28 at.% of Cr can be dissolved in the μ phase (Nb_7_Ni_6_) at this temperature, and the solubility of chromium in NbNi_3_ is approximately 5 at.%. Under these circumstances the low-temperature (cubic) modification of the NbCr_2_ Laves phase can dissolve up to 6 at.% of nickel, but further increase of the Ni content (up to approximately 10 at.%) stabilizes the hexagonal (high-temperature) modification of the Laves phase. The presence of this pseudo-ternary compound which is in equilibrium with all binary intermetallics and body-centred cubic (BCC) Nb- and Cr-based solid solutions largely determines the topology of the isotherm at 1,100 °C. The formation of this phase was also observed in the reaction zone between Nb and Ni–Cr solid solution when chromium concentration exceeded 15 at.%.

**Graphical abstract:**

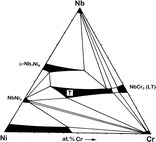

## Introduction

The practical interest in the Nb–Ni–Cr system is generated mainly by the fact that Ni–Cr-based alloys are important corrosion-resistant high-temperature materials. Their application in combination with the Nb-based alloys having a very high specific strength at elevated temperatures might lead to a significant improvement in the high-temperature performance of various structural components. To optimize the fabrication procedure of such composite structures, it is paramount to be able to predict (and control) the phases which are formed at the interfaces between niobium and Ni–Cr alloys upon solid-state bonding and under service conditions. It was repeatedly demonstrated that the optimal starting point for research on any metal–metal interactions is the investigation of the phase equilibria and reactive phase formation in relevant materials systems.

The Nb–Ni–Cr system has been studied extensively within a limited composition domain bounded by the binary NbNi_3_ and NbCr_2_ intermetallics and Ni- and Cr-based solid solutions [1–7]. Most of the cited work was performed almost 50 years ago. In the investigated composition region the partial liquidus projections, several isothermal sections (in the temperature range 1,100–1,200 °C) and several isopleths have been established. For more details and more complete bibliography the reader should access a rather exhaustive compilation by Gupta [8].

On the contrary, for the higher Nb-containing regions of this system only very limited experimental information about phase relations is available [9]. Therefore the present investigation was designed to establish the phase relations in the Nb–Ni–Cr system at 1,100 °C (below the liquidus temperature [4]) and to gain insight into the morphological evolution of the interfacial region between niobium and Ni–Cr solid solution during the reaction at this temperature.

## Results and discussion

### Phase equilibria in the Nb–Ni–Cr system at 1,100 °C

The isothermal cross section through the ternary diagram was constructed by the traditional method of equilibrated alloys and diffusion couple technique. Diffusion couples with two-phase end-members were used. The efficiency of this technique in constructing isothermal cross sections through ternary phase diagrams is higher compared with that when single-phase alloys are used as end-members of the couples because in this case, the chance to “hit” interfaces at which three phases are in equilibrium is much larger. Further details concerning the use of diffusion couple techniques in studying phase diagrams can be found in Ref. [10].

**Fig. 1:**
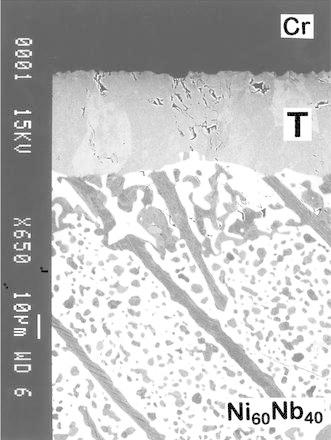
Back-scattered electron image (BEI) showing the morphology of the diffusion zone developed between chromium and two-phase alloy with the nominal composition Ni_60_Nb_40_ after annealing in vacuum at 1,100 °C for 196 h. Note that the NbNi_3_ domains within the microstructure of the two-phase end-member exhibit a *white contrast*

The microstructure of the reaction zone developed after annealing (1,100 °C, 196 h) in the diffusion couple based on pure Cr and two-phase alloy with nominal composition Ni_60_Nb_40_ consisting of (after the equilibration) μ-Nb_7_Ni_6_ intermetallic and NbNi_3_ is given in Fig. 1. From the micrograph one can notice that a continuous layer of pseudo-ternary phase (henceforth designated as “T”) is a dominant reaction product in this diffusion couple. The product ternary compound appears to be in equilibrium with Cr-based solid solution as well as with both constituents of the initial two-phase alloy. The latter indicates a three-phase equilibrium NbNi_3_ + μ-Nb_7_Ni_6_ + T in the Nb–Ni–Cr system at this temperature.

**Fig. 2:**
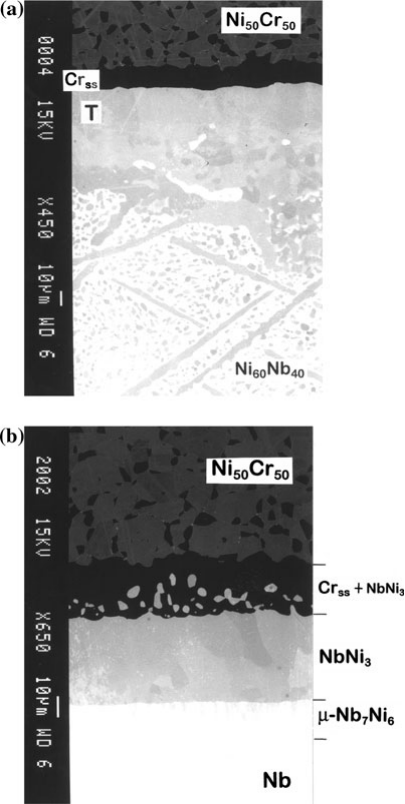
BEIs of the reaction zones developed in the annealed (1,100 °C, 196 h) diffusion couples based on two-phase alloy with the nominal composition Ni_50_Cr_50_ and **a** the two-phase alloy Ni_60_Nb_40_ and **b** pure Nb. Note that domains of the Cr-based solid solution (*Cr*_*ss*_) present within the microstructure of the Ni_50_Cr_50_ alloy exhibit a *dark contrast* (the various phases on the micrographs are denoted by their binary formulae, and *T* is the pseudo-ternary phase)

When another two-phase material with the nominal composition Ni_50_Cr_50_, which after equilibration at 1,100 °C and quenching in water is a mixture of face-centred cubic (FCC) Ni-based and body-centred cubic (BCC) Cr-based solid solutions [8], was used as the end-member of the diffusion couple, the interfacial reaction with the Ni_60_Nb_40_ alloy also resulted in the formation of a continuous layer of the pseudo-ternary intermetallic (Fig. 2a). An interesting feature here is that the product layer of the T phase is not in direct contact with the initial two-phase substrate, but separated from it by the layer of Cr-based solid solution. This implies that in the Nb–Ni–Cr system the pseudo-ternary compound T is not in equilibrium with the Ni-based solid solution at 1,100 °C.

There also exists an equilibrium between Cr-based solid solution and NbNi_3_ compound on the Nb–Ni–Cr isotherm. This can be appreciated from the diffusion zone morphology developed in a Ni_50_Cr_50_/Nb couple after reaction at 1,100 °C for 196 h (Fig. 2b).

From the diffusion couple experiments described above, it may be suggested that on the Nb–Ni–Cr isotherm at 1,100 °C the Cr-based solid solution is involved in three-phase equilibria Cr_ss_ + NbNi_3_ + T and Cr_ss_ + NbNi_3_ + Ni_ss_. Moreover, information obtained with the diffusion technique was used as a guide for selecting the compositions of the alloys used to verify the provisionally found equilibria and to determine the boundaries of the phase fields in this ternary system.

**Fig. 3:**
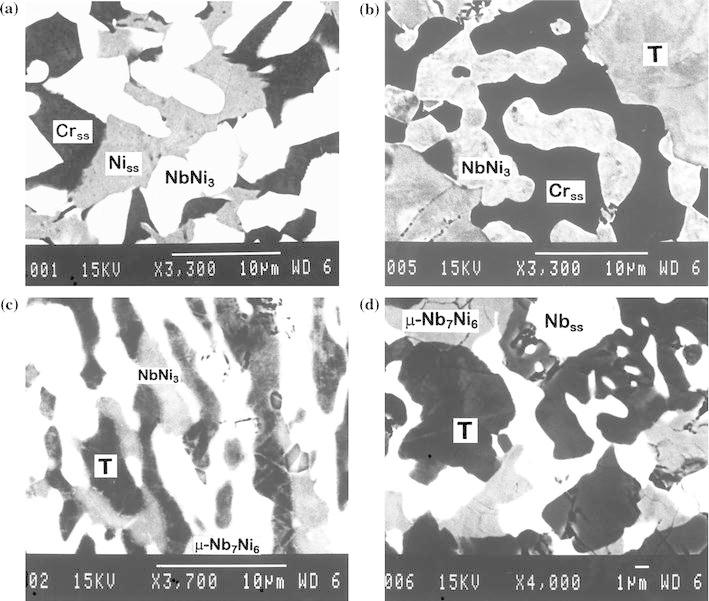
Microstructure of the three-phase alloys of the Nb–Ni–Cr system after equilibrating at 1,100 °C in vacuum for 196 h and quenching **a** Ni_50_Cr_40_Nb_10_, **b** Ni_40_Cr_40_Nb_20_, **c** Ni_55_Cr_5_Nb_40_, and **d** Ni_15_Cr_35_Nb_50_ (the various phases on the micrographs are denoted by their binary formulae. *T* is the pseudo-ternary phase, and *Ni*_*ss*_, *Cr*_*ss*_, and *Nb*_*ss*_ are the Ni-, Cr-, and Nb-based solid solution, respectively)

As it was expected, after equilibrating at 1,100 °C for 400 h and quenching ternary alloys with nominal composition Ni_50_Cr_40_Nb_10_, Ni_40_Cr_40_Nb_20_, Ni_55_Cr_5_Nb_40_, and Ni_15_Cr_35_Nb_50_, they indeed exhibited a three-phase morphology (Fig. 3). The composition of the phases present in the alloys after heat treatment was measured with electron probe microanalysis (EPMA) and the corresponding three-phase equilibria were plotted on the isotherm. It was found that nearly 28 at.% of Cr can be dissolved in the μ phase (Nb_7_Ni_6_) at this temperature, and the solubility of chromium in the NbNi_3_ is approximately 5 at.%.

In order to determine more precisely the phase boundaries on this isotherm a number of equilibrated two- (and single-) phase alloys were studied with optical microscopy, EPMA, and X-ray diffraction (XRD). Results of this investigation are summarized in Table 1.

**Table 1:** Phases present in equilibrated alloys after annealing at 1,100 °C for 400 h and quenching

Alloy	Phases^a^ present at 1,100 °C
Ni_75_Cr_10_Nb_15_	Ni_ss_ + NbNi_3_
Ni_55_Cr_15_Nb_30_	T + NbNi_3_
Ni_30_Cr_5_Nb_65_	μ-Nb_7_Ni_6_ + Nb_ss_
Ni_25_Cr_15_Nb_60_	μ-Nb_7_Ni_6_ + Nb_ss_
Ni_30_Cr_25_Nb_45_	μ-Nb_7_Ni_6_ + T
Ni_30_Cr_45_Nb_25_	Cr_ss_ + T
Ni_15_Cr_60_Nb_25_	Cr_ss_ + T
Ni_3_Cr_77_Nb_20_	Cr_ss_ + NbCr_2_ (LT)
Ni_3_Cr_57_Nb_40_	Nb_ss_ + NbCr_2_ (LT)
Ni_6_Cr_60_Nb_34_	NbCr_2_ (LT)
Ni_8_Cr_58_Nb_34_	NbCr_2_ (LT) + T
Ni_10_Cr_50_Nb_34_	T

*T* pseudo-ternary phase, *Ni*_*ss*_, *Cr*_*ss*_, and *Nb*_*ss*_ Ni-, Cr-, and Nb-based solid solution, respectively

^a^The various phases are denoted by their binary formulae

With respect to the last three alloys listed in the table some comments concerning the phase determination have to be made. It turned out to be extremely difficult to draw a definite conclusion about the stability region of the pseudo-ternary phase in the Nb–Ni–Cr system at 1,100 °C on the basis of the results of XRD analysis. Attempts to discriminate between the XRD patterns produced by these intermetallics were inconclusive. Therefore, a thorough investigation using polarized light microscopy has been performed. Three ternary alloys with a fixed Nb content of 34 at.%, namely Ni_6_Cr_60_Nb_34_, Ni_8_Cr_58_Nb_34_, and Ni_10_Cr_50_Nb_34_, were annealed at 1,100 °C and examined with EPMA, XRD, and polarized light microscopy. The alloy with the nominal composition Ni_6_Cr_60_Nb_34_ turned out to be a single-phase material and does not show any polarization effect, which is not a surprising finding given the fact that the low-temperature modification of the NbCr_2_ Laves phase of the binary Nb–Cr system possesses a cubic structure of the MgCu_2_ type (C15, *cF*24) [8]. On the other hand, the Ni_8_Cr_58_Nb_34_ alloy after equilibration and quenching exhibits two-phase morphology. It was observed that some domains within the microstructure corresponding to the pseudo-ternary phase show a distinct polarization effect, which indicates a crystal symmetry lower than cubic.

The third alloy Ni_10_Cr_50_Nb_34_ also appears to be a single-phase material, but contrary to the Ni_6_Cr_60_Nb_34_ alloy, differently oriented grains of the optically anisotropic T phase within the microstructure show distinctly different colours in white polarized light due to reflection pleochroism.

As to the nature of the phase T in the Nb–Ni–Cr system at this temperature, it is well known that in some instances the crystal structure of a Laves phase is dependent on electron concentration [11] and in the case of the Nb–Ni–Cr system it was conjectured that nickel can stabilize the high-temperature (HT) modification of NbCr_2_. This is consistent with the results of the present work; at 1,100 °C the low-temperature (LT) cubic modification of the NbCr_2_ Laves phase can dissolve up to 6 at.% of nickel, whereas further increase of the Ni-content (up to approximately 10 at.%) stabilizes the HT (hexagonal MgZn_2_ type, C14, *hP*12) modification of the Laves phase.

**Fig. 4:**
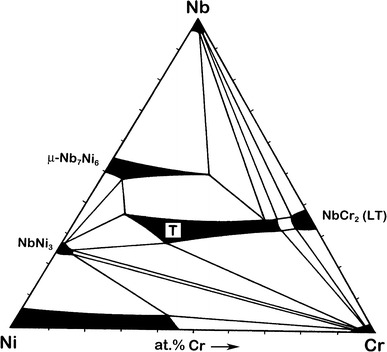
Isothermal cross section through the ternary phase diagram of Nb–Ni–Cr at 1,100 °C determined in the present study

Finally, the results from phase analysis and concentration measurements in diffusion couples and equilibrated alloys lead to the cross section of the Nb–Ni–Cr phase diagram at 1,100 °C represented in Fig. 4.

### Reactions at the interfaces between Nb and Ni–Cr solid solution alloys at 1,100 °C

**Fig. 5:**
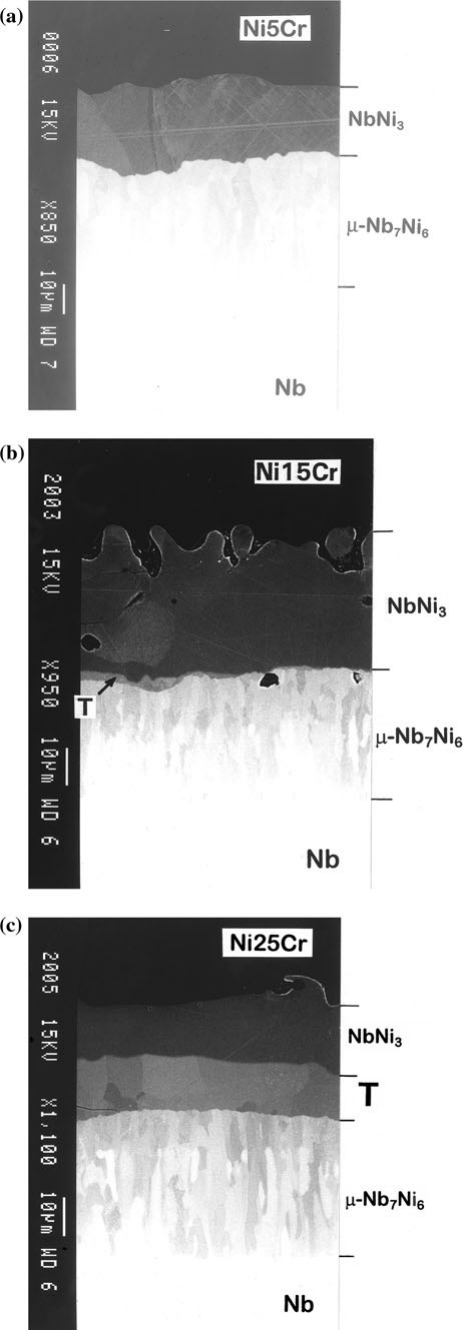
BEIs of the reaction zones developed in the diffusion couples based on Nb and Ni–Cr solid solution alloys with different Cr content **a** 5 at.%, **b** 15 at.%, and **c** 25 at.%

The microstructure of the reaction zones in the diffusion couples based on niobium and various Ni–Cr solid solution alloys are shown in Fig. 5. Solid-state reaction of Ni–5 at.% Cr solid solution with Nb at 1,100 °C results in reaction products somewhat similar to those that would be expected (from the phase diagram) in a binary Nb/Ni couple [8]. Two binary intermetallic compounds, viz. NbNi_3_ and μ-Nb_7_Ni_6_ with very low chromium content (<0.3 at.%), are formed in the transition zone (Fig. 5a). It was found that the reaction layer growth follows a parabolic kinetics profile which is indicative of a diffusion-controlled process.

However, when 15 at.% of Cr was introduced into nickel, interaction in the diffusion couple with Nb led to a different morphology. A new feature here is the formation of a continuous (very thin) layer of pseudo-ternary compound T between the NbNi_3_ and μ-Nb_7_Ni_6_ product phases (Fig. 5b). It was observed that at this temperature, diffusion zones of the Ni–Cr/Nb couples containing from 15 to 25 at.% of Ni exhibit similar reaction pattern, although the relative volume of the pseudo-ternary compound within the reaction product increases with increasing the Cr content in the initial end-member (Fig. 5c). Another characteristic feature of the diffusion zones developed in these samples is a significant enrichment of chromium in the Ni-based solution in the vicinity of the alloy–reaction product interface. This implies that in the phase field of the Ni-based solid solution on the ternary Nb–Ni–Cr isotherm the diffusion paths proceed in the direction of increasing chromium concentration before entering the single-phase region of NbNi_3_. This is connected with the higher affinity of Ni towards niobium as compared to that for chromium and mass balance requirements.

## Experimental

Nickel (99.98 %), chromium (99.95 %), and niobium (99.98 %) supplied by Goodfellow (Huntingdon, UK) were used as initial materials. Ni–Cr alloys (5–50 at.% of Cr) were melted in an arc furnace under argon atmosphere using a non-consumable tungsten electrode. The ingots were cold-rolled to a thickness of 1.5 mm. Slices of 8 × 8 mm^2^ were cut from the sheets and homogenized under 1 bar of gas mixture Ar + 10 vol% of H_2_ (H_2_O ≤5 ppm) at 1,100 °C for 100 h.

The various Ni–Cr–Nb alloys were also made in an arc furnace. The ingots of ternary alloys were re-melted five times to improve their homogeneity. The weight loss of the alloys after melting was less than 1 wt% relative. The specimens were annealed in an electro-resistance tube furnace in evacuated quartz ampoules at 1,100 °C. The temperature was controlled within ±3 °C. After annealing the samples were quenched in water.

The diffusion couples were prepared and heat-treated in a vacuum furnace (ca. 5 × 10^−6^ mbar) under an external load of approximately 2 MPa. Temperature control was carried out within ±2 °C accuracy. For a typical experiment, the cooling rate of the sample in the vacuum furnace was about 600 °C/h.

After annealing and standard metallographic preparation the diffusion couples and equilibrated alloys were examined by optical microscopy, scanning electron microscopy (SEM), and EPMA. Owing to the coarse structure of the annealed alloys, XRD analysis was performed with a cylindrical texture camera using nickel-filtered K_α_Cu radiation.
